# The Opening Year

**Published:** 1894-01

**Authors:** 


### Editorial.

### THE OPENING YEAR.

Good resolutions and the ordinary platitudes are indulged in every New Year with an honest prolixity that seems to argue for good not only to the individual, but for society in general. While every good resolution has in it the germs of an incentive to a higher life, and may lead in that direction, it is, unfortunately, too true that in the majority of cases they are written upon the sands of time, to be obliterated by each recurring wave of the world's ever-changing life.

While this is true, the daily and yearly inspiration given is a good one. It is typical of that inner aspiration that is ever looking towards progress, and through its impelling influence civilization advances, professions develop, and man takes the measure of his own abilities and capabilities of meeting the ever-increasing demands of his age.

The New Year is a period—a very brief one, as time is measured, but an appropriate one to moralize, to review the past and build for the future.

The years are not many since the first man essayed the dental art on American soil. More than a century has flown by, but centuries are mere periods, dots on the pages of a world's history; yet this short lapse of time means much to our profession. In that period it has developed beyond the expectations of those who bravely attempted its earliest work. To-day it is recognized as one of the important aids in the amelioration of human ills.

Retrospection is not always profitable, for it brings with it the reflection that however good the work may have been, its imperfections stand out in bold relief, bringing the sense with it of crude manipulations if not positive ignorance. It is probably true that no one who has practised our profession for fifty years but has felt humiliated by his experience. It has been a structure builded upon mistakes, necessarily so, for it was an attempt to form a conception from fractional experiences in many directions. It was an effort to graft mechanical ideas upon medical practice, to make a unique profession, combining the old theories with new ideas. That this met with opposition is not surprising. The old professions treated the new aspirant with contempt. It was regarded as a trade; the assumption of professional equality was looked upon as a feeble effort to elevate it above pure empiricism. This feeling was the natural antagonism of conservatism and had its period, to give place to a more enlarged conception of the value of this labor.

It has taken more than a century to reach the present standard, and it will cover more than the same period to crown the work thus far accomplished. We have no reason for self-complacency. The past has developed much, but we are very far removed from the time when the eighteen thousand men in this country can be called a thoroughly trained body of dentists imbued with the recognized professional spirit, without which a profession cannot exist.

The New Year of 1894 brings with it the impressive fact that in six years the century will have closed its work, the deeds, good and bad, will have passed into history, and the new era of a twentieth century will have begun its record. We cannot deal with it. That it will be a period of wonderful progress, who can doubt? Science, art, and a profounder knowledge of things material will, doubtless, make that century, to those who live through it, more remarkable than any that have preceded it.

We glory in our own century. It has advanced the world's knowledge as no other. The social, material, and spiritual life of the nations has developed beyond all former eras, and as its last years are fleeting by we may feel that, with all its deficiencies, its record will remain as the most brilliant epoch in history.

As a profession we cannot count by centuries, but must look to the passing years for marks of progress. If these have not shown an educating force in developing a better professional life, then may we well despair. We need much. To rest satisfied with our educational facilities, with our standards, preliminary and collegiate, augurs but poorly for advancement. There are no stopping-places in a profession. It is trite to say that a permanent camp means stagnation, yet it embodies a truth which cannot be too often repeated. The various agencies that have strengthened professional work must be used with more power. The indifference must give place to increased activity, and activity should demonstrate its value by results. Each and every one should take part in associative efforts. The load of inertia now carried must give place to an ever-increasing interest. The selfish side of life should change to the better altruistic conception that the labor of each is necessary to make the perfect whole.

Let, then, the New Year open with a resolve that laggardism shall cease; that the profession, like a man's country, needs the labor, voice, and pen of every individual in it, and that this should be given to the extent of ability, without reserve and with enthusiasm. If this resolution be inscribed in indelible characters upon the life of each individual, then the New Year will prove better than preceding ones, and each in its turn will add lustre to the professional life of the century.

